# The gene expression profiles of induced pluripotent stem cells from individuals with childhood cerebral adrenoleukodystrophy are consistent with proposed mechanisms of pathogenesis

**DOI:** 10.1186/scrt130

**Published:** 2012-10-04

**Authors:** Xiao-Ming Wang, Wing Yan Yik, Peilin Zhang, Wange Lu, Patricia K Dranchak, Darryl Shibata, Steven J Steinberg, Joseph G Hacia

**Affiliations:** 1 Department of Biochemistry and Molecular Biology, University of Southern California, 1425 San Pablo Street, Los Angeles, CA 90089, USA; 2 Department of Pathology, University of Southern California, 1441 East Lake Avenue, Los Angeles, CA 90033, USA; 3 Hugo W. Moser Research Institute at Kennedy Krieger, 707 North Broadway, Baltimore, MD 21205, USA

## Abstract

**Introduction:**

X-linked adrenoleukodystrophy (X-ALD) is a complex disorder with variable expressivity that affects the nervous, adrenocortical and male reproductive systems. Although *ABCD1 *mutations are known to provide the genetic basis for X-ALD, its pathogenesis is not fully elucidated. While elevated very long chain fatty acid (VLCFA) levels in blood and reduced VLCFA catabolic activity in cultured fibroblasts are biomarkers used to identify *ABCD1 *mutation carriers, the roles peroxisomal lipid metabolism play in disease etiology are unknown.

**Methods:**

Primary skin fibroblasts from two male patients with the childhood cerebral form of the disease (CCALD) caused by *ABCD1 *frameshift or missense mutations and three healthy donors were transduced with retroviral vectors expressing the *OCT4, SOX2, KLF4 *and c-*MYC *factors. Candidate induced pluripotent stem cells (iPSCs) were subject to global gene expression, DNA methylation, DNA copy number variation, and genotyping analysis and tested for pluripotency through *in vitro *differentiation and teratoma formation. Saturated VLCFA (sVLCFA) and plasmalogen levels in primary fibroblasts and iPSCs from healthy donors as well as CCALD patients were determined through mass spectroscopy.

**Results:**

Skin fibroblasts from CCALD patients and healthy donors were reprogrammed into validated iPSCs. Unlike fibroblasts, CCALD patient iPSCs show differentially expressed genes (DEGs) relevant to both peroxisome abundance and neuroinflammation. Also, in contrast to fibroblasts, iPSCs from patients showed no significant difference in sVLCFA levels relative to those from controls. In all cell types, the plasmalogen levels tested did not correlate with *ABCD1 *mutation status.

**Conclusion:**

Normal *ABCD1 *gene function is not required for reprogramming skin fibroblasts into iPSCs or maintaining pluripotency. Relative to DEGs found in fibroblasts, DEGs uncovered in comparisons of CCALD patient and control iPSCs are more consistent with major hypotheses regarding disease pathogenesis. These DEGs were independent of differences in sVLCFA levels, which did not vary according to *ABCD1 *mutation status. The highlighted genes provide new leads for pathogenic mechanisms that can be explored in animal models and human tissue specimens. We suggest that these iPSC resources will have applications that include assisting efforts to identify genetic and environmental modifiers and screening for therapeutic interventions tailored towards affected cell populations and patient genotypes.

## Introduction

X-linked adrenoleukodystrophy (X-ALD) is a complex disorder caused by mutations in the *ABCD1 *gene that encodes an integral peroxisome membrane protein belonging to the ATP-binding cassette transporter superfamily [1-4]. X-ALD primarily affects the nervous system, adrenal cortex and testes with highly variable clinical presentations that are influenced by modifier genes and the environment [2,3]. Males with *ABCD1 *mutations develop childhood cerebral ALD (CCALD) about 33% of the time and adult onset adrenomyeloneuropathy (AMN) about 45% of the time [2,3]. CCALD patients typically show symptoms between five and nine years of age with rapid cerebral demyelination and adrenocortical atrophy. Within a few years of onset, they suffer dementia and progressive neurological deficits that eventually lead to death. In contrast, AMN patients show a later onset of disease (20 to 40 years of age) and present with adrenal insufficiency, a distal axonopathy in the spinal cord and peripheral neuropathy that results in progressive spastic paraparesis with debilitating end stage disease [2,3]. Approximately 10% of hemizygotes develop primary adrenocortical insufficiency (Addison's disease) with no evidence of nervous system dysfunction [2,3]. Disease prognosis is challenging since mutations do not correlate with clinical phenotypes [5] and male siblings with the same *ABCD1 *mutation, including monozygotic male twins [6,7], can have dramatically different clinical presentations [8]. Although hemizygotes typically show the most severe clinical manifestations of disease, about half of female *ABCD1 *mutation carriers develop AMN-like symptoms later in life [2,3].

The molecular mechanisms underlying the inflammatory brain demyelination found in CCALD patients are not fully understood. It has been hypothesized to be related to the accumulation of saturated very long chain fatty acids (sVLCFAs) in specific central nervous system (CNS) cell types (for example, oligodendrocytes and microglial cells) and/or lipid classes (for example, ganglioside, phosphatidylcholine and cholesterol ester fractions) [9-11]. Other hypotheses have focused on the roles of oxidative stress [12-16], myelin sheath integrity [17], oligodendrocyte apoptosis and microglial cell activation [1,2], and CNS cell membrane receptors [18].

Here, we report the generation and genomic characterization of CCALD patient-specific induced pluripotent stem cell (iPSC) model systems that can provide a platform to investigate cell autonomous processes relevant to X-ALD pathogenesis. The gene expression and biochemical profiles of these patient-specific iPSCs provide a novel perspective that supports the leading hypotheses regarding disease pathogenesis. Furthermore, our resources provide a first step required for the development and interpretation of patient-specific model systems that investigate genetic modifiers, environmental risk factors and non-cell autonomous processes relevant to the etiology of X-ALD.

## Materials and methods

### Cell culture conditions

Primary dermal fibroblast cultures from CCALD patients and controls were obtained from the Peroxisomal Disease Laboratory at the Kennedy Krieger Institute and Coriell Institute Cell Repositories, respectively. All cells described herein were cultured at 37°C with 5% CO_2_. Human primary dermal fibroblasts and mitomycin inactivated mouse embryonic fibroblasts (MEFs) were cultured in fibroblast media (DMEM supplemented with 10% FBS, L-glutamine, penicillin/streptomycin, vitamin solution, and essential and nonessential amino acids (Life technologies, Foster City, CA, USA)), as previously described [19]. iPSCs were cultured on a layer of mitomycin C-inactivated MEF feeder cells in iPSC medium (DMEM:F12 medium supplemented with 20% KSR, L-glutamine, penicillin/streptomycin, nonessential amino acids, β-mecaptoethanol and bFGF (Life technologies, Foster City, CA, USA)) [20,21].

### Cell reprogramming

Five different pMX retroviral vectors designed to deliver green fluorescent protein (GFP) and human *OCT4, SOX2, KLF4 *and *C-MYC *cDNA sequences were obtained from Addgene (Cambridge, MA, USA) [20,21]. Primary human fibroblasts were twice transduced with a mixture of all five retroviruses as described [20,21]. Transduction efficiency was evaluated by GFP expression. After four days, cells were re-plated onto MEF feeders and cultured in hESC medium containing 1 mM valproic acid [22]. By four weeks, candidate iPSC colonies were manually picked and clonally expanded [20,21]. A full list of the analyses conducted on each of the candidate iPSCs is described below and provided in Additional file 1.

### Protein pluripotency biomarker analysis

Alkaline phosphatase staining was performed using the leukocyte alkaline phosphatase kit (Sigma-Aldrich, St. Louis, MO, USA). For immunostaining, cells were fixed in 4% paraformaldehyde for 20 minutes, permeabilized with 1% Triton X-100 for 5 minutes except for surface marker staining, and blocked in 1% BSA in 1 × PBS for 1 hour at room temperature. Primary antibody staining was performed at 4°C overnight with antibodies against OCT4 and NANOG (goat polyclonal IgG against human, R&D Systems, Minneapolis, MN, USA), SOX2 (goat polyclonal IgG against mouse, rat and human) and SSEA4 (mouse monoclonal IgG_3 _against human, Santa Cruz Biotechnology, Santa Cruz, CA, USA), TRA-1-60 (mouse monoclonal IgM against human, Millipore, Billerica, MA, USA), TuJ1 (rabbit monoclonal IgG_1 _against mammalian, Covance Research Products, Princeton, NJ, USA), α-SMA (mouse monoclonal IgG_2a _against human, mouse and so on, Sigma-Aldrich, St. Louis, MO, USA), and AFP (mouse monoclonal IgG_2a _against human, pig and canine, Life technologies, Foster City, CA, USA). Secondary antibody staining was performed at room temperature for one hour with appropriate fluorescence conjugated secondary antibodies from Life technologies, Foster City, CA, USA and Jackson ImmunoResearch, West Grove, PA, USA. Nuclei were visualized by staining with 100 ng/ml DAPI (Life technologies, Foster City, CA, USA).

### Gene expression profiling

Total RNA samples (100 ng per sample) were converted into biotin-labeled cRNA targets (Affymetrix GeneChip^® ^IVT Labeling Kit, Santa Clara, CA, USA), processed and analyzed on Affymetrix Human Genome 133A 2.0 or 133 Plus 2.0 GeneChips, as previously described [19]. Using WebArray software (San Diego, CA, USA), we applied the RMA algorithm to generate log2-transformed gene expression values and linear model statistical analysis (limma) to identify differentially expressed genes (DEGs) with false discovery rates (FDRs) calculated using the spacings LOESS histogram (SPLOSH) method [23,24] (Additional file 2). We performed hierarchical clustering analysis using Partek Genomics Suite software (St. Louis, MO, USA). We conducted GeneOntology (GO) and Kyoto Encyclopedia of Genes and Genomes (KEGG) pathway analyses using WebGestalt software (Nashville, TN, USA) [25-27]. We used the DAVID (Database for Annotation, Visualization and Integrated Discovery) v6.7 bioinformatics resource [28] for the annotation of gene functions. Scaled gene expression scores and .cel files are available at the National Center for Biotechnology Information (NCBI) Gene Expression Omnibus (GEO) repository under Series Accession Number GSE34308.

### DNA methylation profiling

Genomic DNA was extracted from cultured cells as described [29,30] and analyzed on 450 K Infinium Methylation BeadChips (Illumina, San Diego, CA, USA), which interrogate the methylation status of over 485,000 CpG sites distributed across the human genome. The resulting data were analyzed using GenomeStudio software (that is, unmethylated to fully methylated) for each locus (Additional file 3). Bisulfite DNA sequencing was conducted as previously described [29,30].

### Genotyping and copy number analysis

Genomic DNA from cultured cells was also analyzed using Human CytoSNP-12 Infinium HD BeadChips (Illumina) that interrogate the genotypes of 299,140 human single nucleotide polymorphisms (SNPs). Data filtering and analysis were performed in GenomeStudio (Illumina). Copy number analysis was performed using CNVPartition version 2.4.4 with a confidence threshold set at 50 and a minimum of 10 SNP probes per CNV region, as previously described [31]. In multiple samples, we performed the global genotyping analysis two independent times and only assigned a copy number change (CNC) if both analyses were in agreement (Additional file 4). Dideoxysequencing of *ABCD1 *exons 1, 8 and 9 was performed as previously described [32,33].

### *In vitro *differentiation and teratoma assays

iPSCs were detached from culture dishes with collagenase IV, maintained in suspension to induce embryoid body (EB) formation and subjected to an *in vitro *differentiation procedure, as described [34,35]. For teratoma analysis, iPSCs from a confluent 10 cm^2 ^plate were harvested and subcutaneously injected to the dorsal flanks of immunodeficient (SCID) mice (Jackson Laboratory, Bar Harbor, ME, USA), as described [34]. Nine weeks after injection, teratomas were excised, fixed in 10% formalin, sectioned and stained with hematoxylin and eosin.

### Lipid analysis

We used liquid chromatography-tandem mass spectrometry (LC-MS/MS) to measure C26:0-lysophosphorylcholine and phosphatidylethanolamine (PE) plasmalogen levels in cell lysates processed by methanol extraction as described in reference [36]. Herein, C26:0-lysophosphorylcholine measurements were used to evaluate VLCFA levels. The tetradeuterated analog of 1-O-hexadecyl-2-lysn-sn-3 phosphorylcholine was used to quantify PE plasmalogens. PE plasmalogens were identified based on the fragmentation patterns reported in reference [37].

## Results

### Derivation of candidate iPSCs from CCALD patient fibroblasts

**Figure 1:**
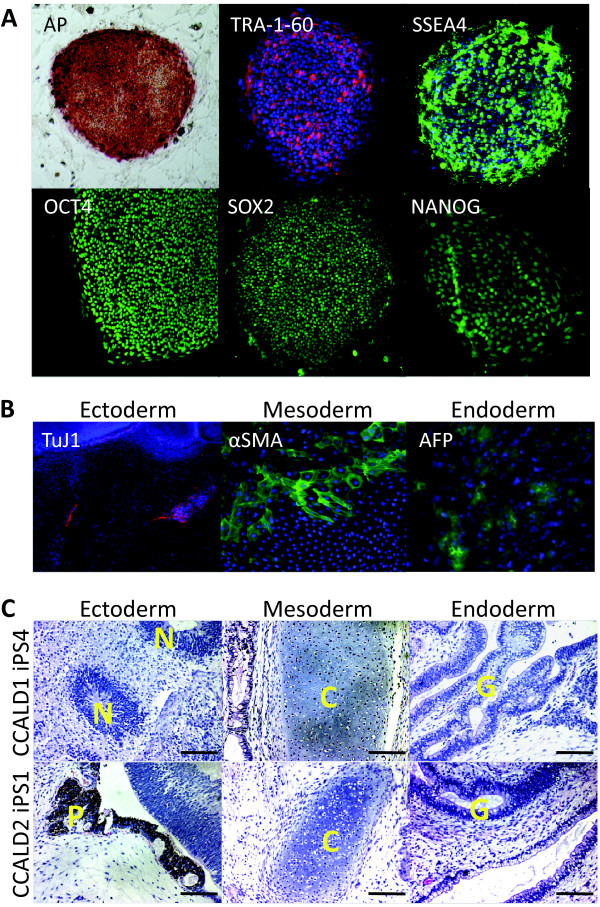
**Characterization of iPSCs from patient with CCALD**. **(A) **Representative images of alkaline phosphatase (AP) staining and pluripotency protein biomarker immunostaining analysis of childhood cerebral adrenoleukodystrophy (CCALD) patient induced pluripotent stem cell (iPSCs) are presented. **(B) **Representative CCALD iPSCs were subject to *in vitro *differentiation (Materials and methods) and cell lineages derived from all three germ layers were identified by immunostaining, as depicted. Antibodies against Tuj1, αSMA and AFP were used to identify cells of ectodermal, mesodermal, and endodermal origin, respectively. **(C) **Histological analysis of teratomas derived from representative CCALD iPSC clones from two patients is provided. Evidence of cell lineages derived from all three germ layers is provided. C, Cartilage tissue; G, Glandular tissue; N, Neural rosettes; P, pigment neuroepithelium. Scale bar = 50 μm.

Primary skin fibroblast cultures from three healthy donors and two CCALD patients (Table 1) were infected with retroviruses designed to express the human *OCT4, SOX2, KLF4 *and *c-MYC *genes (Methods). We observed iPSC-like colonies for two weeks and clonally expanded TRA-1-60 positive colonies for four weeks, consistent with prior reports of reprogramming skin fibroblasts from healthy human donors [38]. All candidate iPSC colonies maintained the expected morphological features and expressed protein biomarkers of pluripotency (Figure 1A, Additional file 1).

**Table 1:** Skin fibroblast and iPS cell donor information

Fibroblast ID	Prior ID	Status	iPS cell ID#	Genotype
**Control1**	AG05838	Healthy female, age 36 yr, passage 6 for reprogramming	Control1-iPS1 Control1-iPS2 Control1-iPS3 Control 1-iPS4	Presumed wild type
**Control2**	AG09599	Healthy female, age 30 yr, passage 8.5 for reprogramming	Control2-iPS1 Control2-iPS2 Control2-iPS3 Control2-iPS4	Presumed wild type
**Control3**	AG13153	Healthy male, age 30 yr, passage 9.5 for reprogramming	Control2-iPS1	Presumed wild type
**CCALD1**	216212	Male CCALD patient Age 9 yr, passage 8 for reprogramming	CCALD1-iPS1 CCALD1-iPS2 CCALD1-iPS3 CCALD1-iPS4	hemizygous *ABCD1* c. 253_254insC; p.P84
**CCALD2**	306463	Male CCALD patient Age 11 yr, passage 8 for reprogramming	CCALD2-iPS1	hemizygous *ABCD1* c.1847C > T; p.A616V

### Genotypes and DNA copy number profiles of iPSCs

We confirmed that the patient iPSCs had the expected mutant *ABCD1 *genotypes and that control iPSCs lacked these specific *ABCD1 *mutations by dideoxysequencing. As determined by BeadArray analysis, the genotypes of over 290,000 SNPs in iPSCs and original fibroblasts were > 99.9% concordant. Based on the same genotyping data, we did not detect copy number changes (CNCs, that is, insertions or deletions greater than 10-kb in length) in patient CCALD1-1, CCALD1-2 and CCALD2-1 iPSCs or Control1-3, Control1-4 and Control2-1 iPSCs (Additional file 4). Consistent with prior reports of reprogrammed human cells [39-41], we detected CNCs in 8/14 (57%) iPSCs analyzed (Additional file 4). These iPSCs had one (CCALD1-3, CCALD1-4 and Control2-3), two (Control1-2 and Control3-1), three (Control2-2 and Control2-4) or five (Control1-1) separate genomic regions affected by a CNC (Additional file 4).

### Gene expression profiles of CCALD and control donor cells

**Figure 2:**
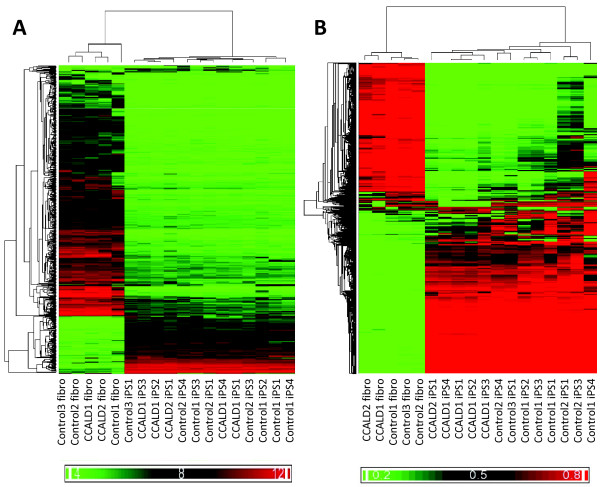
**The gene expression and DNA methylation profiles of fibroblasts and iPSCs**. **(A) **A dendrogram generated based on the unsupervised hierarchical clustering analysis of gene expression data from childhood cerebral adrenoleukodystrophy (CCALD) patient and control fibroblasts and induced pluripotent stem cells (iPSCs) is provided. The analysis was based on data from 672 probe sets producing log2-tranformed gene expression scores with coefficient of variation (CV) > 0.25 and conducted using average linkage and Euclidean distance; **(B) **A dendrogram generated based on the unsupervised hierarchical clustering analysis of DNA methylation data from CCALD patient and control fibroblasts and iPSCs is provided. Data from 7,493 DNA methylation assays interrogating autosomal CpG loci with CV > 0.5 and the fifth largest and smallest β-values being > 0.6 and < 0.4, respectively, were used in this analysis in order to represent the most variable loci. Clustering was performed using average linkage and the Pearson dissimilarity distance.

We validated the robust expression of previously reported iPSC signature genes in control and CCALD donor-derived iPSCs and skin fibroblasts [38] based on a subset of the data generated from global expression profiling of over 18,000 transcripts (Figure 2A). Unsupervised hierarchical clustering analysis based on the expression of preselected pluripotency biomarkers or the most variable transcripts (that is, coefficient of variation (CV) > 0.10 across all samples)) produced two distinct clusters consisting of skin fibroblasts and the iPSCs (Figure 1 and Additional file 5).

### DNA methylation profiles of CCALD and control donor cells

We performed global DNA methylation analysis interrogating over 485,000 CpG sites of all starting fibroblast cultures and reprogrammed iPSCs (Table 1) (Methods). Hierarchical clustering analysis demonstrated that the iPSCs and fibroblasts have distinct DNA methylation profiles that were independent of *ABCD1 *mutation status (Figure 2B).

### *In vitro *differentiation of CCALD patient iPSCs and *in vivo *teratoma formation

All control and CCALD patient-derived iPSCs subjected to *in vitro *embryoid body assays produced cells derived from all three germ layers (Methods) (Figure 1B). Furthermore, all three iPSCs tested (one control and two CCALD patient donors) formed teratomas when injected into immune-deficient mice (Methods). In all these teratomas, histological analysis demonstrated the presence of tissues representative of all three germ layers (Figure 1C).

### Comparative lipid profiling of cells from healthy donors and those with CCALD

**Figure 3:**
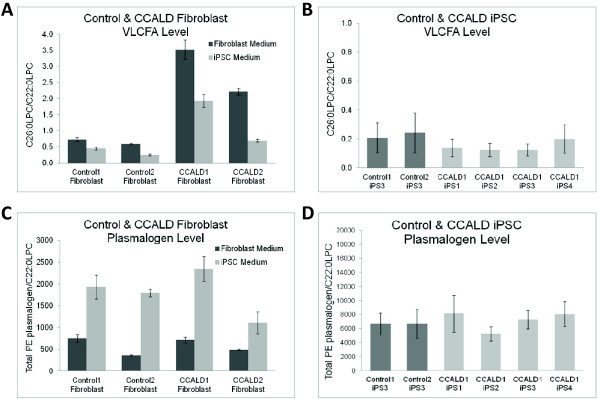
**Comparison of saturated very long chain fatty acid (sVLCFA) and plasmalogen levels in patient and healthy control fibroblasts and iPSCs**. **(A) **Relative sVLCFA levels in patient and healthy control fibroblasts grown in fibroblast media or iPSC media, as represented by the C26:0/C22:0 LPC (lysophosphatidylcholine) ratio; **(B) **Relative sVLCFA levels in patient and healthy control iPSC clones grown in induced pluripotent stem cell (iPSC) media, again represented by the C26:0/C22:0 LPC ratio; **(C) **Relative plasmalogen level in patient and healthy control fibroblasts grown in fibroblast media and iPSC media, as represented by the total phosphatidyl ethanolamine (PE) plasmalogen/C22:0 lysophosphatidylcholine (LPC) ratio; **(D) **Relative plasmalogen in patient and healthy control iPSC clones grown in iPSC media, again represented by the total PE plasmalogen/C22:0 LPC ratio.

Consistent with prior reports [42], cultured CCALD patient skin fibroblasts grown in fibroblast growth media showed 4.3-fold elevated VLCFA levels (based on C26:0-lysophosphorylcholine measurements, see Materials and methods), but similar PE plasmalogen levels relative to fibroblasts from healthy donors (Figure 3A, C)). Similarly, we found 3.8-fold elevated VLCFA levels, but comparable PE plasmalogen levels, in patients relative to control fibroblasts grown under the same conditions in iPSC media (Figure 3A, C). In contrast, no significant differences were found for either VLCFA or PE plasmalogen levels in patient and control iPSCs cultured under the same conditions in iPSC media (Figure 3). Nevertheless, all fibroblast cultures had approximately two-fold elevated PE plasmalogen levels when grown in iPSC relative to fibroblast growth media. The fact that iPSCs cannot be maintained in the undifferentiated state in fibroblast media precluded lipid analyses under these conditions.

### Differential gene expression among patient and control cells

Among the two patient and three control fibroblasts used for reprogramming, no differentially expressed genes (DEGs) ( > 1.2-fold change, FDR < 0.1) were found. This was not unexpected due to the limited number of samples analyzed. To enhance our ability to detect potential DEGs, we conducted a larger-scale gene expression analysis ( > 47,000 transcripts) using cultured skin fibroblasts from five healthy control donors and five CCALD patients. In these studies, we identified 127 DEGs (44 with lower and 83 with higher expression in patient relative to control fibroblasts) (Additional File 6).

Based on GeneOntology (GO) analysis, we found a total of 13 functional categories enriched (≥ 4 genes, B-H corrected *P *< 0.05) for DEGs with higher expression in patient relative to control fibroblasts (Additional file 7). Of these, the most specific GO category was nuclear lumen genes. KEGG analysis did not show any enriched categories (≥ 4 genes, B-H corrected *P *< 0.05) for DEGs with higher expression in patient relative to control fibroblasts. In contrast, DEGs with lower expression in patient relative to healthy control fibroblasts were enriched for one KEGG category ('Pathways in Cancer' as represented by the *SMAD3, LAMA4, PML *and *DAPK1 *genes), but no GO categories.

Given the possible gaps in public databases of gene functions relevant to peroxisome biology and X-ALD pathogenesis, we used the DAVID Bioinformatics resource to annotate the function of DEGs and manually searched for genes relevant to peroxisome biology, lipid metabolism, oxidative stress and neuroinflammation (Additional file 8). The only peroxisomal gene was *AGPS*, which is involved in plasmalogen (a type of ether phospholipid) biosynthesis, and it had higher expression in patient fibroblasts. Nevertheless, we note that the PE plasmalogen levels in patient and healthy control fibroblasts were similar under both growth conditions (Figure 3C). Two gene encoding enzymes involved in the Lands cycle, deacylation/reacylation reactions responsible for glycerophospholipid remodeling [43], were present with the higher expression of *LYPLA1 *(deacylation), but lower expression of *MBOAT7 *(reacylation), in patients relative to control fibroblasts. Two DEGs were involved in sphingosine metabolism with *SMPD1*, which converts sphingomyelin to ceramide, showing lower expression in patients relative to control fibroblasts and *SGPL1*, which degrades sphingosine-1-phosphate, showing higher expression in patients relative to control fibroblasts. This is of interest given the differences in the sphingolipid composition of white matter from the brains of CCALD and healthy individuals [44]. No genes involved in classic oxidative stress responses were found in the list. The DEGs strongly or primarily related to neuroinflammation included *CBLB, RAB27A *and *TCF3*, which showed higher expression in patient fibroblasts, as well as *CD81, MASP1 *and *GRK5*, which showed higher expression in control fibroblasts.

Based on our manual curation, we found that the identity of approximately 40% of the DEGs (46 total) were consistent with the expression profiles of cultured fibroblasts related to the site of skin biopsy (Additional file 6). All these genes showed the highest variability in expression based on biopsy sites, as described in reference [45]. We also note that the expression profiles of 4/6 DEGs described above as being involved in neuroinflammation, (*RAB27A, CD81, MASP1 *and *GRK5*), are also influenced by the biopsy site. Although all the fibroblasts in our study were obtained from the upper limbs, the control and patient donor cells were collected and expanded at different laboratories (Methods), which could influence their gene expression signatures.

We identified 75 DEGs (22 with higher and 53 with lower expression in patient relative to control cells) based on the gene expression profiles of five CCALD iPSCs from two CCALD donors and nine control iPSCs from three healthy donors (Additional file 9). There was no overlap with the Affymetrix probe IDs of the DEGs uncovered in the cultured skin fibroblasts from the five healthy controls and five CCALD patient donors discussed above. Different Affymetrix probe IDs interrogated the *CEP57 *gene indicated it was a DEG in both systems, but in opposing directions (that is, higher in patient relative to control fibroblasts, but higher in control relative to patient iPSCs). Based on GO analysis, we found a total of 14 functional categories enriched (≥ 4 genes, B-H corrected *P *< 0.05) for DEGs with higher expression in patient relative to control cells (Additional file 10). These included blood vessel morphogenesis, regulation of cellular protein metabolic process and carboxylic acid metabolic process. In contrast, GO analysis identified no enriched categories for DEGs with higher expression in healthy control cells. KEGG analysis did not identify any enriched pathways (≥ 4 genes, B-H corrected *P *< 0.05) for DEGs with higher expression in either the patient or control cells.

**Figure 4:**
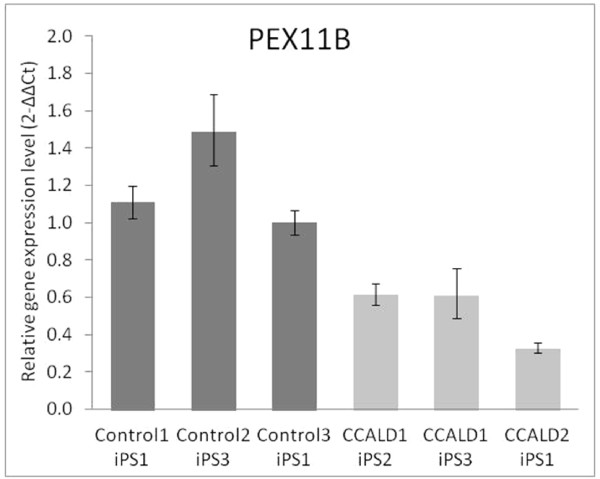
**qRT-PCR confirmation of *PEX11B *gene expression in patient and healthy control iPSCs**. Data from the analysis of *PEX11B *gene expression levels are provided. The error bars represent one standard deviation. The expression was lower in patients relative to control cells (two-tailed Student's *t*-test *P*-value < 0.5).

Although GO and KEGG analysis did not highlight biological processes proposed to be relevant to disease, inspection of the DEG functions based on the DAVID Bioinformatics resource (Additional file 11) uncovered genes associated with major hypotheses pertinent to X-ALD pathogenesis. Among the relevant genes with reduced expression in CCALD patient relative to healthy donor-derived iPSCs were *PEX11B *(confirmed by qPCR in Figure 4) and *CD200*. The former plays a pivotal role in peroxisome proliferation and maintenance [46,47]. Decreased *CD200 *expression is associated with the activation and accumulation of macrophages, including brain microglia, and causes inflammatory responses in other systems [48,49]. DEGs with higher expression in patient relative to control iPSCs were also related to hypotheses relevant to X-ALD pathogenesis and lipid metabolism. *ULK1 *is the mammalian homolog of the yeast *Atg1 *gene, which plays a critical role in the autophagy-mediated turnover of peroxisomes in yeast [50]. *PLA2G2A *is involved in phospholipid turnover [51]. *NAAA, THBS1 *and *BSG *all have functions related to neuroinflammation [52-61]. *SLC7A8 *is a transporter of thyroid hormones, which can induce peroxisomal biogenesis and β-oxidation as well as the *ABCD2 *expression, whose induction can correct biochemical functions of X-ALD patient fibroblasts [62].

### Robust differences in DNA methylation frequently found between fibroblasts and iPSCs are not associated with *ABCD1 *mutation status

In our global DNA methylation analysis (Methods), the starting 5 fibroblasts and 14 iPSCs showed over 62,000 loci where there was a 0.25 unit difference in average β-values and B-H corrected *P *< 0.05 (Additional file 12). To focus on the most robust differentially methylated loci (DML), we identified 744 sites that were hypomethylated (β < 0.2) in all samples of one group and hypermethylated (β > 0.8) in all samples in the remaining group (fibroblasts and iPSCs). We identified 266 distinct genes proximal to at least one DML with approximately half of them (49%) being poorly expressed ( < 60^th ^percentile) in all samples regardless of its methylation status, consistent with our prior results involving the co-analysis of DNA methylation and gene expression profiles in normal and cancerous B-cell populations [29,30]. Nevertheless, 60 genes (23%) were at least modestly higher expressed when the proximal CpG locus was in the hypomethylated relative to hypermethylated state ( > 1.5-fold change, FDR < 0.1 and expressed > 60^th ^percentile in the hypomethylated state).

In contrast, we only found five robust DMLs in comparisons of two patient and three control fibroblasts and one robust DML in comparisons of five patient and nine control iPSCs (Additional file 13). The DML present in the iPSC analysis was also present in the fibroblast analysis, with higher methylation being found in all CCALD patients relative to control donor cells regardless of *ABCD1 *mutation status. This shared DML was proximal to the *PRDM15 *gene, whose expression was not interrogated in our global GeneChip gene expression assays. The remaining four DMLs in fibroblasts were proximal to the *PAX3, CCDC140, UTRN *and *BAIAP2 *genes. All three of the genes interrogated by our GeneChip expression assays (*PAX3, UTRN *and *BAIAP*) were poorly expressed in all fibroblasts regardless of *ABCD1 *mutation status.

### Local gene expression is not substantially affected by CNCs found in iPSCs

To begin to address the influence that CNCs present in iPSC have on their transcriptome, we focused on the expression profiles of genes residing in the affected genomic regions. A total of 11 amplified segments containing 22 unique genes were found in 8 iPSCs. Only six of these unique genes showed elevated expression in the amplified relative to the diploid samples (that is, a > 1.2-fold increase relative to the mean expression scores of diploid samples and falling outside their range). This included the *ID1 *gene in CCALD1-3, *WWC1 *gene in CCALD1-4, and *IQCA1, CXCR7, SQLE *and *KIAA0196 *genes in Control1-1.

Three iPSCs (Control2-2, Control2-4 and Control3-1) showed evidence of having at least one genomic deletion, with evidence in each case that one allele was retained. Collectively, five unique genes were present in the four deleted genomic regions in these iPSCs. There was no evidence of reduced expression in the samples with reduced copy number (that is, > 1.2-fold decrease relative to the mean expression scores of diploid samples and falling outside their range).

### Amplified or deleted segments show no differences in DNA methylation status

A total of 745 DNA methylation assays interrogated loci located within amplified regions present in control or patient iPSCs. In all cases, the DNA methylation status of such genomic regions was similar regardless of whether it was in the diploid or amplified state. In fact, we observed no evidence of a block of DNA methylation change associated with a CNC (that is, three or more contiguous assays wherein the β-value of the amplified segment was greater than 0.2 units outside the range of the diploid samples).

Next, we accessed the methylation status of genomic regions subject to a loss of copy number in iPSCs. A total of 79 DNA methylation assays interrogate loci with the genomic regions of heterozygous deletion. The affected samples included Control2-iPS2 (chr13:q14.2), Control2-iPS4 (chr2:q33.3) and Control3-iPS1 (chr3:p14.2 and chr5:p15.2). Again, we observed no evidence of a block of DNA methylation change associated with a CNC (that is, three or more contiguous assays wherein the β-value of the deleted segment fell more than 0.2 units outside the range of the diploid samples).

## Discussion

X-ALD is a complex peroxisomal disorder with variable expressivity. Although its primary genetic basis has been known for some time [1-4], the exact nature of X-ALD pathogenesis and its genetic and environmental modifiers have not been elucidated. Here, we generated iPSC resources for the longer-term purpose of developing novel tissue culture models for elucidating the pathogenesis of X-ALD and screening for more effective drug therapies. In keeping with prior reports [63], skin fibroblasts from *ABCD1 *mutation carriers can be reprogrammed to form iPSCs with the hallmark molecular properties of pluripotency, including the expression of appropriate gene and protein biomarkers and changes in DNA methylation levels, as summarized in Additional file 1. Patient iPSCs could be differentiated into embryoid bodies and differentiated *in vitro *into representative cell types of all three germ layers. Most importantly, patient iPSCs formed teratomas with evidence of cell types from all three germ layers.

Consistent with prior reports [64-66], we identified *de novo *CNCs over 10-kb in length in approximately half of our iPSCs. These CNCs were only found in healthy donor iPSCs and not those generated from patient cells (Additional file 4). The observed genomic deletions always provided evidence of affecting only one allele and genomic amplifications always involved a limited increase in copy number (three to four copies maximum). Due to the fact that we conducted global expression and DNA methylation analyses on these samples, we could investigate the effects that these CNCs have on the expression of genes located within affected genomic segments. In almost all circumstances, their expression levels were within the range of diploid samples. Although multiple factors likely contribute to these observations, we favor the explanation that this primarily reflects the effects of selection whereby CNCs are only tolerated in iPSCs if they involve genomic regions that do not influence the initiation of reprogramming or maintenance of pluripotency.

As a result of our genomic characterization of these cell resources, we acquired global gene expression data from patient and control fibroblasts. Many DEGs were previously reported to be associated with the site of biopsy [45]. This is reasonable given that the patient and control fibroblasts were acquired from different institutions even though all biopsies involved the upper limbs of donors. We sought to determine if there was enrichment for functional categories or biological processes in the DEGs, keeping in mind the limitations of using cultured cells to study complex diseases involving interactions between multiple organ systems. Only very broad functional categories or KEGG pathways were highlighted in these analyses, with none of them showing a direct relation to disease.

Since there are likely to be gaps in public databases of processes relevant to peroxisome biology and X-ALD pathogenesis, we conducted a manual inspection of gene annotations provided by the DAVID bioinformatics resource and found multiple DEGs involved in immune related processes, but only two (*CBLB *and *RAB27A*) of these genes were not associated with the site of biopsy. *CBLB *plays a critical role in antigen-induced immune tolerance and *Cblb*-deficient mice immunized with myelin basic protein are more susceptible to experimental autoimmune encephalomyelitis (EAE), a model for multiple sclerosis [67,68]. *RAB27A *mutations can lead to an uncontrolled T lymphocyte and macrophage activation syndrome in humans, with some individuals showing possible leukocyte brain infiltration [69]. In one Saudi Arabian kindred, *RAB27 *mutations were associated with immunodeficiency and progressive demyelination of brain white matter [70].

The DEGs found in patient and control iPSCs did not overlap with those found in fibroblasts and instead were consistent with several leading hypotheses regarding X-ALD pathogenesis. This suggests that the reprogramming process can minimize the confounding influence the site of skin biopsy has on the gene expression profiles of cultured fibroblasts. In particular, we highlight the reduced expression of *PEX11B*, a major controller of peroxisome proliferation and neuroinflammatory genes, in patient relative to control iPSCs. *Pex11B *null mice show several pathologic features, including neuronal migration defects, enhanced neuronal apoptosis, developmental delay, hypotonia and neonatal lethality [71]. Despite these severe phenotypes, *Pex11B *null mice displays only mild defects in peroxisomal fatty acid beta-oxidation and ether lipid biosynthesis [71]. Intriguingly, the deletion of a single *Pex11B *allele leads to a slightly increased number of peroxisomes, increased levels of oxidative stress in brain tissue, and neuronal cell death in mice [72]. In addition, the *ULK1*, whose yeast homolog plays a critical role in the autophagy-mediated peroxisome turnover [50], showed higher expression in CCALD patient relative to control iPSCs. It is tempting to speculate that if nervous and immune system cells of *ABCD1 *mutation carriers indeed have aberrant PEX11B and ULK1 activity, this could lead to differences in peroxisome abundance and/or activity that increase reactive oxygen species (ROS) levels and promote X-ALD pathogenesis. It remains to be determined if *ABCD1 *mutation carriers have abnormal peroxisome abundance in their pertinent nervous and immune system cells and tissues.

In a similar vein, the increased *NAAA, THBS1, BSG *(*aka CD147 aka EMMPRIN*) and *NOTCH1 *gene expression in patients relative to control iPSCs is supportive of hypotheses regarding a predisposition to neuroinflammation that is a prelude to devastating autoimmune responses. NAAA hydrolyzes palmitoylethanolamide (PEA), a naturally occurring lipid amide that, when administered as a drug, inhibits inflammatory responses [56]. In principle, increasing leukocyte NAAA levels could reduce PEA levels and promote inflammation. In fact, a chemical inhibitor of *NAAA *function attenuates inflammation and tissue damage and improves recovery of motor function in mice with spinal cord trauma [56]. Intriguingly, CD200 has been proposed to play a role in the immune privileged status of the CNS when CD200-mediated immune suppression occurs via neuron-microglial as well as glial-glial interactions in inflammatory conditions [73]. *THBS1 *is linked to neuroinflammatory processes involving astrocyte and microglia through its role in processing and activating the TGF-β ligand [74] and is also implicated in responses to oxidative stress [75]. Likewise, *Notch1 *is involved in microglial associated inflammation [76]. Also of relevance are emerging reports that *BSG *acts a master regulator of matrix metalloproteinases (MMP) implicated in most diseases involving neuroinflammation and thus has been proposed to play a role in the immune-privileged status of the CNS [77].

Although we highlight the possible implications of the gene expression profiles observed in patient iPSCs, we note alternative hypotheses regarding their origins and biological significance. While the iPSCs described in this study have the hallmark properties of pluripotency, their gene expression profiles could reflect subtle *ABCD1 *mutation status-dependent differences in their predisposition to differentiate into specific cell types and lineages. Comparisons of the gene expression profiles of mature cell types derived from patient- and healthy donor-derived iPSCs will be especially informative. The persistence or elimination of groups of DEGs reflective of biological processes and pathways could provide a means of assessing the tissue specificity of disease and enhance the ability to discern biologically informative gene expression signatures from noise resulting from confounding variables, such as tissue culture conditions.

Although *ABCD1 *mutation carriers show elevated sVLCFA levels in their blood and urine and reduced sVLCFA catabolic activity in their cultured fibroblasts, the role of sVLCFA in disease pathogenesis is still under discussion. The significance of decreased plasmalogen levels in the patients' brain white matter also is unclear [44,78]. As expected, CCALD patient fibroblasts had elevated VLCFA levels, but similar PE plasmalogen levels, relative to those from healthy donors (Figure 3A, C). Likewise, iPSCs from CCALD patient and healthy control donors also showed similar PE plasmalogen levels (Figure 3D). The fact that all patient and control iPSCs tested had low VLCFA levels, based on C26:0-lysophosphorylcholine measurements, (Figure 3B) is puzzling, yet consistent with prior reports [63]. VLCFA levels are determined by their rate of synthesis (that is, catalyzed by fatty acid elongating enzyme ELOVL1 [79]), degradation (affected by ABCD1 and ABCD2 function [80]) and uptake of these fatty acids from the culture medium. As such, one hypothesis is that the rate of VLCFA synthesis is lower in iPSCs relative to fibroblasts under the culture conditions evaluated. Consistent with this hypothesis and prior reports [63], the expression of the *ELOVL1 *gene was significantly lower (2.6-fold, FDR < 2.2 × 10^-9^) in iPSCs relative to fibroblasts. Nevertheless, we note that *ELOVL1 *was not differentially expressed in patient relative to control fibroblasts or iPSCs. An alternate hypothesis that the *ABCD2 *gene is compensating for the impaired *ABCD1 *function in patient iPSCs; however, *ABCD2 *was not differentially expressed in patient relative to control fibroblasts or iPSCs. This does not preclude the possibilities that ABCD2 activity is being increased on the protein level or that another gene is playing a major role in significantly lowering VLCFA levels in CCALD iPSCs. We also note a prior hypothesis that the rapid growth rate of iPSCs could reduce their VLCFA levels, independent of their *ABCD1 *mutation status [63]. Fibroblasts have altered morphology and slowed growth in iPSC media relative to fibroblast media, which according to the growth rate hypothesis could contribute to their reduced VLCFA levels. Given that iPSCs can rapidly differentiate in fibroblast media, iPSC growth media provides an imperfect, but necessary, compromise in direct comparisons between cultured fibroblasts and iPSCs. We note the potential contribution of MEF feeder cells to iPSC lipid profiles and the advantages of using feeder-free media in future experiments.

### Future applications and directions

The impending implementation of newborn screening for X-ALD based on blood lipid profiles will increase the demand for model systems to screen for more effective therapeutic interventions [36,81]. Early detection would provide physicians with a window of opportunity to treat presymptomatic patients prior to the development of CCALD, and may also prevent or delay AMN onset. Therapeutic interventions, such as Lorenzo's Oil, help prevent the onset of cerebral disease in some individuals, but are not effective for the majority of CCALD patients and, likewise, there are no effective options for AMN [75-77]. A compelling attribute of iPSC model systems is that they represent the exact *ABCD1 *mutations found in the patient population and thus provide an opportunity to test therapeutic agents tailored to a patient's genotype in cell populations most affected by disease. Examples of genotype-dependent therapeutic strategies include nonsense suppressor drugs [33] and molecular chaperones [82] for individuals with nonsense and missense mutations, respectively.

The fact that CCALD iPSCs show gene expression profiles similar to those derived from healthy controls may reflect the fact that X-ALD clinical symptoms do not manifest at birth but, instead, occur in early childhood or later in life. Given that *ABCD1 *mutant mice show clinical aspects of X-ALD with increasing age [83], it is possible that later passage CCALD iPSCs and their derivatives may manifest gene expression profiles and/or functional properties more consistent with disease pathogenesis and progression. In this regard, a comparison of the properties of iPSCs and their derivatives previously obtained from other CCALD and AMN patients [63] as a function of *in vitro *passage number could be informative. Despite the promise of iPSC approaches, it will remain a significant challenge to generate and optimize *in vitro *model systems for X-ALD and other complex disorders that involve multiple organ systems as well as unknown gene-environment interactions and genetic modifiers.

## Conclusions

We have reprogrammed skin fibroblasts from CCALD patients and control donor primary fibroblasts into iPSCs that show all the fundamental hallmark molecular and cellular properties of pluripotency. The DEGs found in comparisons of patient- and healthy donor-derived iPSCs are consistent with emerging hypotheses regarding the role of peroxisomes, oxidative stress and neuroinflammation in the pathogenesis of X-ALD. Given that the sVLFCA profiles of the patient iPSCs are similar to those of control iPSCs, these gene expression profiles are not dependent on the presence of the hallmark lipid biomarkers of disease. Additional gene expression and functional analyses involving differentiated cell types derived from CCALD and control iPSCs could be especially informative given our preliminary results. This would include cell types related to the CNS (for example, oligodendrocytes, astrocytes and microglia), adrenocortical and male reproductive (for example, Leydig and Sertoli cells) aspects of disease. Furthermore, investigations involving patient tissue samples and animal models are required in order to determine if the observed fibroblast and iPSC gene expression profiles are reflective of pathogenic mechanisms or are simply specific to our cultured cells.

## Abbreviations

AMN: adrenomyeloneuropathy; CCALD: childhood cerebral adrenoleukodystrophy; CNC: copy number change; CNS: central nervous system; CV: coefficient of variation; DAPI: 4',6-diamidino-2-phenylindole; DAVID: Database for Annotation: Visualization and Integrated Discovery; DEGs: differentially expressed genes; DMEM: Dulbecco's Modified Eagle Medium; DML: differentially methylated loci; EAE: experimental autoimmune encephalomyelitis; EB: embryoid body; ES: embryonic stem; FBS: fetal bovine serum; FDRs: false discovery rates; GFP: green fluorescent protein; GO: GeneOntology; iPSC: induced pluripotent stem cell; KEGG: Kyoto Encyclopedia of Genes and Genomes; LC-MS/MS: liquid chromatography-tandem mass spectrometry; LPC: lysophosphatidylcholine; MEFs: mouse embryonic fibroblasts; MMP: matrix metalloproteinases; NCBI: National Center for Biotechnology Information; PE: phosphatidylethanolamine; PEA: palmitoylethanolamide; ROS: reactive oxygen species; SNPs: single nucleotide polymorphisms; sVLCFA: saturated very long chain fatty acid(s); X-ALD: X-linked adrenoleukodystrophy.

## Competing interests

The authors declare that they have no competing interests.

## Authors' contributions

XMW and WYY derived the iPSCs from patient and healthy donor fibroblast cultures and conducted immunostaining analysis of protein pluripotency biomarkers. XMW conducted all the global gene expression, genetic and epigenetic analyses. PZ assisted XMW in the teratoma analysis and also provided technical advice in cellular reprogramming and maintaining iPSCs. WL provided technical advice in cellular reprogramming and maintaining iPSCs. DS supervised the histological analysis of teratomas and advised XMW in the data analysis. SJS carried out the biochemical analyses in this project. JGH and XMW were involved in the overall design and conception of the project, statistical analysis of all data sets and wrote the manuscript with the help of all the other authors. All authors read and approved the final manuscript.

## Acknowledgements

We thank P. Watkins, G. Raymond, N. Braverman and R. Pelikan for thoughtful discussion and/or assistance in data analysis. We thank D. Weisenberger and D. Van Den Berg at the USC Epigenome Center for conducting the Illumina BeadArray DNA methylation and SNP genotyping assays and for advice in data analysis. This study was funded by the National Institutes of Health (GM072447 and GM072447-S1) and a California Institute of Regenerative Medicine Pre-doctoral Training Grant (WYY).

## Supplementary Material

Additional file 1 **Characterization of candidate induced pluripotent stem cells (iPSCs)**. A complete listing of the protein, genetic, epigenetic, and cell differentiation assays performed on the iPSCs described in this study is provided. Click here for file

Additional file 2 **Log-transformed gene expression scores from childhood cerebral adrenoleukodystrophy (CCALD) patient and healthy donor control fibroblasts and induced pluripotent stem cells (iPSCs)**. A complete list of log-transformed gene expression scores from CCALD patient and healthy donor control fibroblasts and iPSCs is provided. Click here for file

Additional file 3 **DNA methylation assay beta-values from childhood cerebral adrenoleukodystrophy (CCALD) patient and healthy donor control fibroblasts and induced pluripotent stem cells (iPSCs)**. A partial list of DNA methylation assay beta-values from CCALD patient and healthy donor control fibroblasts and iPSCs is provided. Click here for file

Additional file 4 **Molecular karyotyping analysis of induced pluripotent stem cells (iPSCs)**. A full list of copy number changes detected in the iPSCs described in this study is provided. Click here for file

Additional file 5 **Hierarchical clustering analysis gene expression data from childhood cerebral adrenoleukodystrophy (CCALD) patient and control fibroblasts and induced pluripotent stem cells (iPSCs) based on pluripotency genes**. The analysis was based on gene expression data from 30 pluripotency genes reported in reference [41] and performed with average-linkage and Euclidean distance. Click here for file

Additional file 6 **Differential gene expression between childhood cerebral adrenoleukodystrophy (CCALD) patient and healthy donor control fibroblasts**. A complete list of all gene expression scores and differentially expressed genes (DEGs) between CCALD patient and healthy donor control fibroblasts is provided. Click here for file

Additional file 7 **Gene Ontology (GO) analysis of differentially expressed genes (DEGs) between childhood cerebral adrenoleukodystrophy (CCALD) patient and healthy donor control fibroblasts**. GO analysis of DEGs between CCALD patient and healthy donor control fibroblasts is provided. Click here for file

Additional file 8 **The Database for Annotation, Visualization and Integrated Discovery (DAVID) annotation of differentially expressed genes (DEGs) found in childhood cerebral adrenoleukodystrophy (CCALD) patient and control donor fibroblasts**. DAVID annotation of DEGs found in CCALD patient and control donor fibroblasts is provided. Click here for file

Additional file 9 **Differential gene expression between childhood cerebral adrenoleukodystrophy (CCALD) patient and healthy donor control iPSCs**. A complete list of differentially expressed genes (DEGs) between CCALD patient and healthy donor control induced pluripotent stem cells (iPSCs) is provided. Click here for file

Additional file 10 **Gene Ontology (GO) analysis of differentially expressed genes (DEGs) between childhood cerebral adrenoleukodystrophy (CCALD) patient and healthy donor control induced pluripotent stem cells (iPSCs)**. GO analysis of DEGs between CCALD patient and healthy donor control iPSCs is provided. Click here for file

Additional file 11 **Database for Annotation, Visualization and Integrated Discovery (DAVID) annotation of differentially expressed genes (DEGs) found in childhood cerebral adrenoleukodystrophy (CCALD) patient and control donor induced pluripotent stem cells (iPSCs)**. DAVID annotation of DEGs found in CCALD patient and control donor iPSCs is provided. Click here for file

Additional file 12 **Differentially methylated loci (DML) between fibroblasts and induced pluripotent stem cells (iPSCs)**. A complete list of DML between fibroblasts and iPSCs is provided. Click here for file

Additional file 13 **Differentially methylated loci (DML) between patient and control fibroblasts and induced pluripotent stem cells (iPSCs)**. A complete list of DML between patient and control fibroblasts and iPSCs is provided. Click here for file
