# Supplementary material for: The gene expression profiles of induced pluripotent stem cells from individuals with childhood cerebral adrenoleukodystrophy are consistent with proposed mechanisms of pathogenesis
Source: Stem Cell Res Ther. 2012 Oct 4;3(5):39. doi: 10.1186/scrt130 (PMC3580430; doi:10.1186/scrt130)
Supplement: Additional file 5 — Hierarchical clustering analysis gene expression data from childhood cerebral adrenoleukodystrophy (CCALD) patient and control fibroblasts and induced pluripotent stem cells (iPSCs) based on pluripotency genes. The analysis was based on gene expression data from 30 pluripotency genes reported in reference [41] and performed with average-linkage and Euclidean distance. [file scrt130-S5.PPTX]

## Slide 1
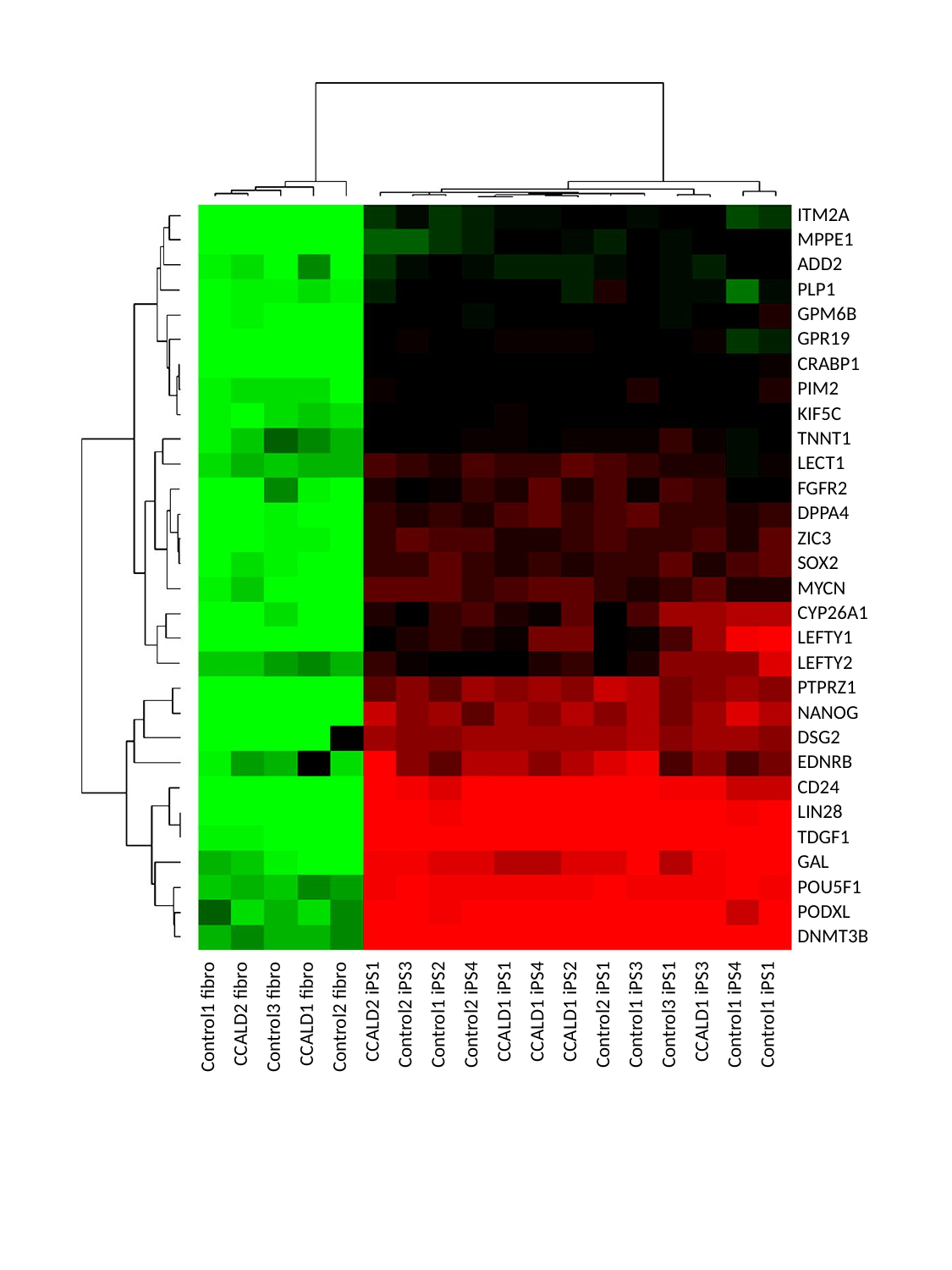

| ITM2A |
| --- |
| MPPE1 |
| ADD2 |
| PLP1 |
| GPM6B |
| GPR19 |
| CRABP1 |
| PIM2 |
| KIF5C |
| TNNT1 |
| LECT1 |
| FGFR2 |
| DPPA4 |
| ZIC3 |
| SOX2 |
| MYCN |
| CYP26A1 |
| LEFTY1 |
| LEFTY2 |
| PTPRZ1 |
| NANOG |
| DSG2 |
| EDNRB |
| CD24 |
| LIN28 |
| TDGF1 |
| GAL |
| POU5F1 |
| PODXL |
| DNMT3B |
| Control1 fibro | CCALD2 fibro | Control3 fibro | CCALD1 fibro | Control2 fibro | CCALD2 iPS1 | Control2 iPS3 | Control1 iPS2 | Control2 iPS4 | CCALD1 iPS1 | CCALD1 iPS4 | CCALD1 iPS2 | Control2 iPS1 | Control1 iPS3 | Control3 iPS1 | CCALD1 iPS3 | Control1 iPS4 | Control1 iPS1 |
| --- | --- | --- | --- | --- | --- | --- | --- | --- | --- | --- | --- | --- | --- | --- | --- | --- | --- |
